# Middlesex Hospital

**Published:** 1829-10

**Authors:** 


### MIDDLESEX HOSPITAL.

In connexion with the preceding case, we may mention that of Crispin Hollings, æt. fifty-three, whom we saw placed on the operating table at the theatre of the Middlesex Hospital, on the 12th of August. We observed a granulating wound in the perineum, and an elastic catheter in the bladder projecting through the rectum. We learnt that, a week before, an attempt had been made by a skilful surgeon to divide a stricture in this man's urethra, where the passage had been long obstructed, to relieve retention of urine. The attempt, however, had failed, and the surgeon had adopted the alternative of puncturing the bladder through the rectum.

Mr. MAYO, under whose care this case at present fell, proceeded to operate in the following way: He passed a moderate-sized silver catheter along the urethra, till it met with resistance at the common seat of stricture, the ligament of Camper. He then with a scalpel opened the half-closed wound in the perineum, and cut upon and exposed the end of the catheter. The catheter was then half withdrawn, and the operator made an incision about half an inch in length from the end of the pervious part of the urethra, in the direction of the natural channel, cutting through the callous substance which formed the stricture, the edge of the knife being turned outwards. Mr. Mayo then tried to pass the catheter into the bladder, but found no passage; upon which he reintroduced the scalpel, making two incisions, one as before, the other beginning at the same point, but with the edge of the knife directed upwards. The catheter then passed readily into the bladder, and was secured there; the elastic instrument being at the same time withdrawn from the rectum.

The patient has gone on uniformly well; nothing has occurred worthy of notice, except a continuance of the passage of urine by the rectum, the opening through which into the bladder is slow in healing.

The operation lasted about three minutes. This is the third case, within twelve months, in which Mr. Mayo has performed this operation at the Middlesex Hospital.

Mr. Mayo observes, that the occasions on which this or any other operation for dividing strictures is necessary in private practice, are extremely rare; while among the poor, who go on suffering severely year after year from stricture, without seeking relief, they are proportionately frequent.

The operation is strictly a dissection. When the end of the catheter has been exposed resting on the stricture, the operator is left to rely entirely upon his anatomical knowledge to enable him to thrust the scalpel in a just direction through the indurated substance into the dilated channel of the membranous portion of the urethra beyond. We are supposing that the stricture with which the operator has to deal is situated in its common place, the ligament of Camper.

Mr. Mayo withdraws the catheter on the second day, and replaces it with another; the catheter is changed every day afterwards. In some cases the silver catheter produces little irritation, and may be used to the end; in others the elastic gum catheter is found to be preferable.

### *Strangulated Crural Hernia, with Omentum adhering to the Sac.*

Rosanna Ainsworth, ætat. forty-four, was first afflicted with crural hernia on the left side in January last; the tumor was easily reducible, and she has worn no truss. On the 16th of August, towards evening, she was attacked with vomiting and purging, and in the night the rupture came down in larger volume than usual, and she could not return it. She was brought to the Middlesex Hospital on the evening of the 17th. At ten o'clock p.m. Mr. Mayo saw her. She was not relieved; what she took into the stomach she vomited; she was alternately chilly and hot, the pulse 120; the belly is large and soft, the abdominal parietes remarkably thin; the tumor is firm and doughy, painful, and extremely sensible to pressure; there is much tenderness of the belly just above the left groin. The patient had been bled and put in the warm bath, where ineffectual attempts were made to reduce the tumor; a large injection had been administered. Under these circumstances Mr. Mayo proceeded to operate forthwith.

The sac having been opened, the stricture, which was uncommonly strict and tense, was relieved by dividing Gimbernat's ligament. A fold of bowel, of a mahogany colour, was then returned. There remained in the sac a portion of thickened omentum, which was very generally adherent to the sac: it was left there, the wound dressed, and the patient put to bed.

No feature of importance occurred during this patient's recovery; but it was thought prudent to apply leeches three several times to the neighbourhood of the umbilicus, at which part the belly for several days remained uneasy and tender on pressure, although the bowels had acted freely in twenty-four hours after the return of the hernia.

The dressings and bandage were removed on the fourth day, and a poultice alone substituted in their place; under which treatment the omentum, which was seen in the wound dark and inclined to slough, granulated, and, as the wound closed, seemed well fitted to form a plug at the neck of the sac, calculated to prevent the return of the rupture.

In a case of scrotal hernia, in which Mr. Mayo operated a twelvemonth ago at this hospital, there was a much larger and an unadherent mass of thickened omentum in the sac. Mr. Mayo removed the thickened part, and returned the rest into the belly, having tied ten or twelve vessels with single threads. The patient recovered, but the hernia has since come down again, and in considerable volume.

---

### *Fungous Tumor of the Lip.*

William Long, æt. fifty-one, for three or four years had observed a wart upon the middle of his under lip: it gave him no inconvenience till about a year ago, when it began to enlarge, and, as it grew, became painful; latterly he suffered nearly constant sensations of darting pain, shooting from the lip to the cheek. Various attempts were made, by the use of escharotics and *partial* excision, to remove the complaint.

This patient came into the Middlesex Hospital on the 11th of August. The tumor was then as large as a middling-sized orange; it was a soft and spongy fungus, and grew from an extensive base, consisting of half the breadth of the under lip, the angle of the mouth, and a third of the breadth of the upper lip. The substance of the lip and cheek immediately adjoining the tumor was extremely indurated.

On the morning after this patient's admission, the tumor bled profusely, which led Mr. Mayo to determine upon its immediate removal. This was effected by cutting through the cheek and lips, at about one third of an inch from the base of the fungus. The flesh at this distance from the fungus was firmer and harder than natural; but Mr. Mayo considered it as thickened from irritation only, not as forming part of the disease.

The operation left a large gap in the face, the edges of which were supported by adhesive plaster and bandages; for it seemed impossible to bring the cut surfaces into any manner of contact. The case has gone on uniformly well, and it is quite surprising, as the wound has cicatrised and contracted, how small the deficiency in the under lip appears.

